# Pulmonary Hypertension: Updates in Diagnosis and Management

**DOI:** 10.3390/jcm14072400

**Published:** 2025-03-31

**Authors:** Estefania Oliveros, Anil Jonnalagadda, Anjali Vaidya

**Affiliations:** 1 Department of Cardiology, New York Presbyterian Hospital/Columbia University Medical Center, New York, NY 10032, USA; 2 Division of Cardiovascular Disease, Department of Medicine, Temple University Hospital, Philadelphia, PA 19140, USA

Pulmonary hypertension (PH) management requires thoughtful evaluation from the clinicians. This is a new era of new diagnostic criteria, medications, and modifications of the risk stratification [1]. In this edition we provide the reader with an overview of updated data for practical implications and incorporation in their daily practice. We have collected relevant studies from investigators that work within this space.

Over the last 2 centuries, medicine has rapidly evolved and the medical landscape has expanded fast, leading to the evolution of specialization and sub specialization. As we amplify our knowledge with also focus more to create clinical and research advances in specific areas. When we examine the data on right heart failure mortality, large amount of interest in reduction of mortality and heart failure hospitalization has led to multiple clinical trials and new medications to improve outcome.

What updates are in this issue?

1. Use of intravenous diuresis in cases of severe precapillary PH, and teach the reader the importance of cautious diuresis in the setting of preload dependence to avoid renal injury and hemodynamic compromise.
2. The significance of partial pressure of carbon dioxide and need to use blood gas analysis in the risk stratification of individual with pulmonary arterial hypertension.
3. Sleep related breathing disorders in patient with chronic thromboembolic pulmonary hypertension, and report the importance of early interventions in this group.
4. A novel finding in obesity and the relationship of VE/VCO2 using invasive cardiopulmonary exercise testing in chronic thromboembolic pulmonary disease.
5. There are 5 reviews that include:
  a. The relationship of pulmonary hypertension and the importance on rhythm control strategies in arrhythmias
  b. The use of exercise testing in the risk assessment of patients with PH
  c. A narrative review of underrepresented minorities in PH, and steps to mitigate disparities
  d. Description of post-capillary PH and practical considerations in the setting of limited and conflicting data
  e. And we finalize with a practical summary for the primary care physician for the diagnostic evaluation of patients with PH

Is there a need?

Pulmonary arterial hypertension patients treated at a Pulmonary Hypertension Care Center accredited by the Pulmonary Hypertension Association have improved survival and fewer hospitalizations. Patients are more frequently prescribed vasodilators, leading to better outcomes. The biggest barriers include monitoring disease progression, complex treatment regimens and side effects of drug-drug interactions. Teaching and sharing our knowledge with the primary care physicians will allow for patients to have expedited diagnostic testing, and referrals.

Consensus statements among pulmonary hypertension experts endorse early and accurate diagnosis and management of PH to improve patient outcomes. Patients often present in late stages, with a rare disease and there is struggle to provide treatment and identify the PH phenotypes in a timely manner.

In the United States, many pulmonologists and cardiologists care for patients with PH, but there are only few dedicated fellowships in this space. We believe in the importance of creating structured curriculums and teach the practicing physicians about the nuances and complexities of PH.
